# The prevalence of metabolic syndrome and associated factors among adults on antiretroviral therapy in Dar es Salaam, Tanzania

**DOI:** 10.21203/rs.3.rs-4372785/v1

**Published:** 2024-05-15

**Authors:** Innocent Yusufu, Tumaini Nagu, Theresia A. Ottaru, Mary Mmwanyika Sando, Sylvia Kaaya, Erasto Mbugi, Lisa R Hirschhorn, Claudia Hawkins

**Affiliations:** Africa Academy for Public Health (AAPH); Muhimbili University of Health and Allied Sciences; Muhimbili University of Health and Allied Sciences; Africa Academy for Public Health (AAPH); Muhimbili University of Health and Allied Sciences; Muhimbili University of Health and Allied Sciences; Robert J. Havey, MD Institute for Global Health, Feinberg School of Medicine, Northwestern University; Robert J. Havey, MD Institute for Global Health, Feinberg School of Medicine, Northwestern University

**Keywords:** HIV, Metabolic syndrome, Antiretroviral therapy, Dolutegravir

## Abstract

**Background:**

Adults living with HIV (ALHIV) are at increased risk of developing metabolic syndrome (MetS). Several factors are associated with an increase in MetS in these individuals, including certain antiretroviral therapies (ART). There is limited data on the prevalence of MetS among ALHIV in sub-Saharan Africa following scale up of newer integrase inhibitor-containing ART regimens.

**Objective:**

We assessed the prevalence and correlates of MetS among ALHIV patients receiving tenofovir, lamivudine, and dolutegravir (TLD) in Tanzania.

**Methods:**

We conducted a retrospective cross-sectional analysis of ALHIV aged ≥18 enrolled in a cardiovascular health study at six HIV Care and Treatment Clinics from 11/2020–1/2021 in Dar es Salaam, Tanzania. MetS was defined according to the National Cholesterol Education Program Adult Treatment Panel III (NCEP ATP III). Descriptive statistics were used to summarize the results, and logistic regression was used to assess demographic, behavioral, and HIV-related risk factors associated with MetS. Covariates with a p-value <0.2 at the univariate level were included in the multivariate model.

**Results:**

Three hundred and eighty nine participants were included in the analysis. The mean age (SD) was 43 years (±11) years, and 286 (73.5%) were female. The prevalence of MetS in this population was 21%. In univariate analysis, MetS components that were significantly higher among women vs. men included abdominal obesity (27.3% vs. 4.9%), reduced HDL (77.9% vs. 53.4%), and elevated glucose (18.5% vs. 14.6%), all p< 0.05. Age≥ 50 yrs [AOR 3.25; (95% CI 1.80–5.84), p < 0.01] and BMI [AOR 0.16; (95% CI 0.09–0.30), P ≤0.01] were both associated with an increased odds of MetS in multivariate analyses.

**Conclusion:**

MetS. is prevalent among Tanzanian ALHIV on TLD. Routine screening for MetS and healthy lifestyle promotion, particularly among women and those aging, should be a priority to prevent against cardiovascular disease. Further studies are needed to monitor the long-term impact of these newer ART regimens on MetS and CVD.

## Background

Approimxately 1.5 million adults are living with HIV (ALHIV) in Tanzania (1) According to the Tanzania HIV Impact Survey 2022–2023, there has been remarkable progress toward the achievement of the UNAIDS 90-90-90 targets in adults, with 94% of people with antiretroviral therapy (ART) and 87% with HIV virologic suppression (2). With improved life expectancy, ALHIV are living longer and are at increased risk of developing non-communicable diseases and other age-related disorders. This includes Cardiovascular diseases (CVD) and associated cardiometabolic risk factors, which are becoming increasingly prevalent in persons with HIV and at a higher rate compared to those without HIV (3).

Metabolic syndrome (MetS) consists of interrelated cardio-metabolic conditions (increased blood pressure, high blood sugar, excess body fat around the waist, and abnormal serum cholesterol or triglyceride levels), which are associated with an increased risk of cardiovascular events, diabetes, and death (4, 5). The burden of MetS is growing globally and is expected to rise substantially over the next few years in low- and middle-income counties (LMICs) where HIV is prevalent (6). MetS is an increasing concern in sub-Saharan Africa (SSA), where the population is steadily aging and experiencing an epidemiologic transition from communicable to non-communicable diseases (NCDs)(5, 7–9). The prevalence of MetS in SSA ranges from 10–30%, with some variation depending on diagnostic criteria (10–13). MetS is also higher among persons with HIV compared to those without HIV (2). In a recent systematic review of studies from SSA, the prevalence of MetS among ALHIV was 2-fold higher compared to persons without HIV [21.5% (95% CI 15.09–26.86) vs. 12.0% (95% CI 5.00–21.00%); p = 0.055], with some heterogeneity across studies (3).

Risk factors for MetS common to both persons with and without HIV include aging, unhealthy behaviors such as a sedentary lifestyle, long working hours, physical inactivity, and diets high in unsaturated fat (14). Chronic inflammation resulting from HIV itself and ART can further increase susceptibility to cardio-metabolic disorders among ALHIV (15). ART has been shown to be associated with abnormalities in the components of MetS, such as elevated triglycerides and reduced high-density lipoprotein (HDL) through various mechanisms (5). Recent research has shown that Integrase strand transfer Inhibitors (INSTI)-based regimens, notably dolutegravir, may be associated with weight gain, obesity, and body fat deposition (16, 17). However, the long-term impact of dolutegravir and other INSTIs on metabolic health is unclear.

In this study we assessed the prevalence of MetS among ALHIV initiating or receiving a combination of tenofovir, lamivudine and Dolutegravir (TLD)-ART in Dar es Salaam, Tanzania. We also determined demographic and clinical characteristics associated with MetS, results of which may be used to help guide public health interventions to better screen for and prevent MetS in this setting.

## Methods

### Study design, population and setting.

We conducted a retrospective cross-sectional analysis among ALHIV enrolled in a longitudinal cohort study examining CVD disease burden and risks *“Ideal Cardiovascular Health: Distribution, Determinants and Relationship with Health Status among People Living with HIV in Urban Tanzania”* which has been described elsewhere (18). This study was conducted in six urban public HIV Care and Treatment clinics (CTCs) in Dar es Salaam, Tanzania. Study participants included ALHIV age ≥18 who were receiving care at these HIV CTCs at least 12 months prior to enrollment. Pregnant women and persons unable to give informed consent were excluded. A total of 629 participants were recruited between November 2020 to January 2021. In this analysis, only participants who were receiving or about to initiate TLD, and those with complete lab results were included (n=389).

### Clinical care in HIV CTCs

HIV care and treatment in Tanzania is provided free of charge under the test and treat approach. TLD is the current recommended first-line regimen for ALHIV which became available in Tanzania in 2019 (19). Patients followed at either monthly clinic visits or every six months if stable, are defined as being on ART for at least six months with no adverse drug reactions that require regular monitoring, good adherence, undetectable HIV viral load< 50 copies/ml and no current illness (opportunistic infections) (19).

Medications for hypertension, diabetes and dyslipidemia are provided free of charge when they are available at the facility. Otherwise, patients are given a prescription to procure (out of pocket payment or covered by health insurance) these medications at a nearby pharmacy. The Tanzania HIV National Guidelines (19), recommend all patients should be screened for CVD risk factors including blood pressure (BP), body weight and height for body mass index (BMI) measurement at each clinic visit, and recieve health education on lifestyle modification to reduce CVD risk. If available at the facility, the guidelines also recommend blood glucose, lipids and chemistry testing every three months to annually depending on avaliable resources.

### Data collection

Data extracted for this analysis included demographics (age, sex, education level, health insurance and occupation), CVD risk factors data, and HIV clinical data (duration on ART, HIV viral load), collected at the first (entry) study visit, using a structured questionnaire administered by a study clinician. Questionnaires were all translated into Swahili. CVD risk factor data included behavioral characteristics such as physical activity, diet, smoking and alcohol consumption, defined based on the American Heart Association (AHA) standard guideline for ideal cardiovascular health index (CVHI) and categorized as being at ideal (1 point), intermediate (0 point) of poor (0 point)(20). (Table 1)

Definition of Ideal cardiovascular health index (Table1)

The ideal diet was adapted from a standard definition of dietary score. Fruits and vegetable intake were used as proxy for diet score (with >20 total servings of fruits and vegetables weekly as the cutoff for ideal intake)(21).

At the entry study visit, participants also underwent anthropometric measurements including, weight waist circumference, height/weight and blood pressure measurement. Weight was measured using a digital scale (Seca, Germany) to the nearest 0.1 kg, and height by a calibrated stadiometer fixed to the wall (Seca, Germany) to the nearest 0.5 cm. BMI was then calculated as weight in kilogram divided by the square if height in metres. A flexible tape measure was used to measure waist circumference (WC) at the level of the iliac crest to the nearest 0.5 cm. Blood pressure (BP) was measured in mmHg to the nearest 1mmHg using a digital sphygmomanometer (Omron). Three BP readings were collected after ensuring the participant was seated in a comfortable position with their arm at the level of the heart. An average of the three readings was computed and considered final. Participants also had blood collected for fasting blood glucose levels, measured using a capillary finger prick test. A standardized automated point of care analyzer (ACCU-CHEK Performa, Roche, Germany) was used. Venous blood was collected to assess the lipid profile including total cholesterol, high density lipoprotein (HDL), low densitiy lipoprotein (LDL) and triglycerides, analyzed using Cobas 400 analyzer (Roche Diagnostics).

### Outcomes

The primary outcome in this study was the prevalence of MetS among ALHIV. MetS was defined according to the NCEP ATP III definition, as present if there were ≥3 of the following five criteria: waist circumference over 40 inches (men) or 35 inches (women), BP≥ 130/85 mmHg, fasting triglyceride (TG) level >150 mg/dl, fasting high-density lipoprotein (HDL) cholesterol level < 40 mg/dl (men) or 50 mg/dl (women) and fasting blood sugar ≥ 100 mg/dl (22).

The NCEP ATP III definition is one of the most widely used criteria of MetS. It incorporates key features of hyperglycemia/insulin resistance, visceral obesity, atherogenic dyslipidemia and hypertension. It uses measurements and laboratory results that are readily available to healthcare providers, hence facilitating its clinical and epidemiological application (23).

### Ethical approval

The primary study received ethical clearance from Muhimbili University of Health and Allied Sciences (MUHAS) -MUHAS-REC-01-2023-1500 in Tanzania and Northwestern University, Chicago (STU00218902).

### Statistical methods

Data analysis was conducted using STATA version 16 (STATA Corp Inc., TX, USA). Chi-Square test (Fisher’s exact test) and student t-test were used to compare demographic characteristics by sex. Logistic regression was used to determine the association between MetS and covariates; age, sex, education level, occupation, insurance status, smoking status, alcohol drinking, physical activity, diet, BMI, duration on ART and HIV viral load. Co-variates with effect sizes were significant at P< 0.2 were included in multiple logistic regression models. The odds ratio (OR) was presented with 95% CI and P < 0.05 considered significant.

## Results

### Demographic characteristics of participants

Three hundred and eighty nine study participants on TLD were analyzed [females 74%, 46% ≤ 50 yrs]. The majority of the participants had at least a primary level of education (73.3%), were employed (84.6%) and had no health insurance (85%). Among the participants, 88% were virally suppressed (HIV VL< 50 copies/mL). The mean number of years since starting ART treatment was 6.4 SD ± 4.8 years. Only 25% reported smoking in the past 30 days. Approximately half of the participants (49%) were overweight/obese (Table 2).

### Overall prevalence of Mets and comparison of MetS and MetS components by gender

**Figure 1:**
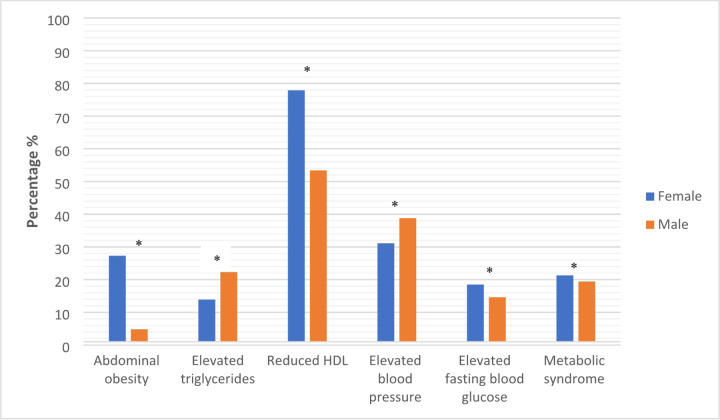
Distribution of cardiometabolic components and metabolic syndrome by gender (23) * p<0.05

The overall prevalence of MetS was (20.82%, n=81). There was a slightly higher prevalance of MetS among females (21.3%, n=61) vs. males (19.4%, n=20) p=0.68. Women had a significantly higher prevelance of abdominal obesity (27.3% vs. 4.9%), reduced HDL (77.9% vs. 53.4%) and elevated glucose (18.5% vs. 14.6%) compared to men. In contrast, men had a higher prevalence of elevated BP (38.8% vs. 31.2%) and higher tryglycerides (22.3% vs. 13.9%), (p values all <0.05) (Figure 1).

### Comparison of MetS and MetS components by age

**Figure 2:**
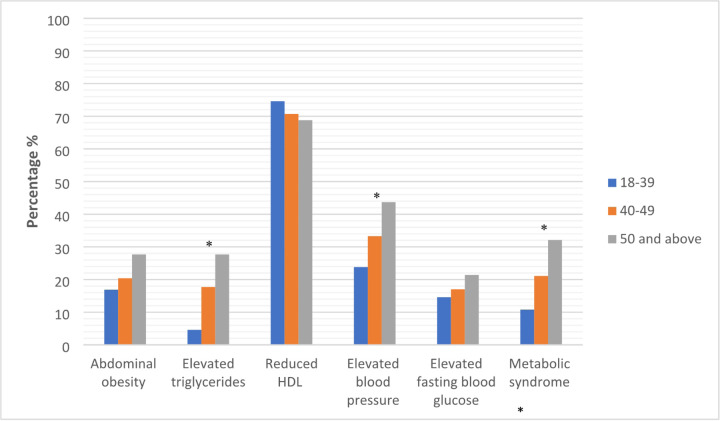
Distribution of cardiometabolic components and metabolic syndrome by age (23) * p<0.05

The prevalance of MetS overall, elevated tryglycerides, abdominal obesity, elevated blood pressure, elevated fasting glucose increased with age category and was highest among participants age ≥50 vs. other age groups. However, statistical significance (p < 0.05) was observed only for MetS overall, elevated triglycerides, and blood pressure (Figure 2).

### Factors associated with MetS

In univariate analyses, age ≥50 yrs, at least primary level of education, alcohol use and physical inactivity was associated with an increased odds of MetS. In multivariate analysis, PLWH ≥50 years were 3.3 times more likely to have MetS vs. other age groups after adjusting for sex, ideal diet, ideal physicial activity, alcohol consumption, duration on ART and HIV viral load. Having an ideal BMI was associated with a 0.16 decreased odds of MetS. There was no association between duration on ART or HIV viral load and MetS either at the univariate or multivariate level. (Table 3)

## Discussion

In this study we report a high prevalence (21%) of MetS among ALHIV on TLD. Similar rates ranging from 19–31% have been reported in other studies conducted in Tanzania and SSA, with variations across each study according to ART regimen type, study populations and MetS diagnostic criteria (10,12,21,24,25). Most of these studies were conducted prior to the rollout of Dolutegravir. Our data suggests MetS remains an important problem among ALHIV in low and middle income settings, even in the era of newer integrase HIV therapies.

We found high proportions of elevated blood pressure, obesity and low HDL levels contributing to MetS, which are already a growing problem among people with HIV in this setting (24). With over 3.2 million people affected with HIV, MetS is expected to rise substantially over the next few years as people age and continue to survive longer on ART. Therefore, there is an urgent need to implement routine assessment and management of cardiometabolic conditions such as weight, lipids, blood pressure and fasting blood sugar into HIV CTCs.

Increasing age and higher BMI were the only risk factors independently associated with MetS similar to several other studies conducted in high and LMICs (26,27). In this study, the prevalence of MetS was also found to increase with age with the highest odds among those aged ≥50. Several studies in both populations with and without HIV have reported an association between excessive body weight gain and MetS (26–28). High BMIs and central obesity are the most common components of MetS in studies from high income countries and SSA (29). It is notable that almost half of the cohort in this study were overweight (BMI ≥25kg/m^2^) with a significantly higher prevalence of overweight and obesity among females compared to males. Results are consistent with those from a large cross sectional community-based study of 6691 participants without HIV in Dar es Salaam (30). Recently, integrase inhibitors (particularly bictegravir and dolutegravir) and tenofovir alafenamide (TAF) have been implicated in weight gain in randomized controlled and observational studies, including in SSA (31).

ALHIV are also at risk of overweight and obesity due to risk factors such as poor dietary habits, alcohol intake and sedentary living (32,33). In this study although the majority of participants were fairly active (ideal physical activitiy 75%), however, ideal diet was very poor (6%) which may have also contributed to the high prevalence of overweight/obesity. Although not a component of MetS, unhealthy diets have been linked to other CVD risk factors such as high blood pressure, elevated blood cholesterol, diabetes, as well as overweight and obesity (34). In East Africa inadequate consumption of fruits and vegetables (the key indicator in determining ideal diet) and high intake of meat has been shown to be associated with increased blood cholesterol and high blood pressure (25). Close monitoring of weight especially among high risk groups such as women are needed, as are strategies to help maintain healthy body weight, lifestyle modification, ideal diets and exercise at the time of ART initiation (35).

Although not statistically significant, a notable other finding in this study was the higher prevalence of MetS among females compared to males. MetS has been reported more commonly in women in several studies from SSA, including those with HIV (3,24,25). Gender specific differences in MetS components observed in this study, including higher prevalence’s of overweight/obesity, reduced HDL and elevated fasting glucose among women, have been similarly reported in other SSA studies (10). In Nigeria, female sex was an independent predictor of all three of these MetS components in both people with and without HIV (36). In Tanzania, over 1.4 million people living with HIV (PLHIV) ages 15 to 64 years are female, stressing the urgent need for screening for MetS components and treatment in HIV clinics in this setting, as well as research to inform on the optimal timing of CVD screening in this vulnerable population.

This is one of very few MetS studies that have been conducted among ALHIV residing in urban Tanzania. The NCEP ATP III criteria is the easiest to apply clinically and epidemiologically standard for defining metabolic syndrome (23). This criteria rely on measurements and laboratory findings that are readily accessible to healthcare professionals, thereby facilitating their practical application in both clinical and epidemiological contexts. The NCEP ATP III only require the presence of at least three out of the five criteria, unlike other criteria such as World Health Organization (WHO) and International Diabetes Foundation which necessitate specific parameters must be present. There were a few limitations which should be noted including the relatively small sample size, which limits generalizability to other settings outside of Dar es Saalam. The cross sectional design of the study limits the ability to establish a true cause and effect relationship between MetS and risk factors assessed. In this study we didn’t collect data on family history of CVD risk factors, some of which have been shown in other studies to be strong predictor of MetS (37). Furthermore, we were not able to obtain information on the date of switch or initiation of dolutegravir based regimen among study participants who had been on prior regimens, and therefore unable to determine exact duration of dolutegravir exposure. However, more than half of persons with HIV in the HIV CTCs included in this study, switched to dolutegravir containing regimens by December 2019.

## Conclusion

In conclusion, we found a substantial burden of MetS among ALHIV on TLD based regimen in urban Tanzania. Our findings align with other studies in the region and highlights the ongoing challenge of MetS among ALHIV, even with the advancement of HIV therapies. As the prevalence of risk factors contributing to MetS continues to rise in ALHIV in Tanzania, the need for routine assessment and management of cardiometabolic parameters in HIV care and treatment clinics is paramount. Tailored interventions and screening strategies may be also needed for women and older persons who remain at higher risk.

## Figures and Tables

**Table:** 

	POOR (0)	INTERMEDIATE (0)	IDEAL (1)
Smoking	Current smoker or have quit ≤12 months ago	Have quit >12 months ago	Never smoked
BMI	≥30 kg/m^2^	25.0–29.9 kg/m^2^	<25 kg/m^2^
Physical activity	None	1–149 min per week of moderate intensity activity and/or 1–74 min per week of vigorous intensity physical activity	≥150 min per week of moderate intensity activity and/or ≥75 min per week of vigorous intensity physical activity
Diet*	None–total servings of fruits and vegetables weekly as the cutoff for ideal intake	1–20 total servings of fruits and vegetables weekly as the cutoff for ideal intake	≥20 total servings of fruits and/or vegetables weekly as the cutoff for ideal intake

**Table 2:** Demographic characteristics of participants

Variable	Male n=103 n (%)	Female n=286 n (%)	Total n=389 n (%)	p-value
**Age group (years)**				
18–39	25 (24.3%)	105 (36.7%)	130 (33.4%)	<0.05*
>=40–49	31 (30.1%)	116 (40.6%)	147 (37.8%)	
>=50	47 (45.6%)	65 (22.7%)	112 (28.8%)	
**Education level**				
**Primary and below**	61 (59.2%)	224 (78.3%)	285 (73.3%)	<0.01*
**Secondary and above**	42 (40.8%)	62 (21.7%)	104 (26.7%)	
**Occupation**				
**Unemployed/retired**	14 (13.6%)	46 (16.1%)	60 (15.4%)	0.11*
**Self-employed**	62 (60.2%)	192 (67.1%)	254 (65.3%)	
**Employed**	27 (26.2%)	48 (16.8%)	75 (19.3%)	
**Insurance status**				
**No insurance**	252 (88.1%)	80 (77.7%)	332 (85.4%)	0.01*

**BMI**				
Underweight (<18.5kg/m^2^)	5 (4.9%)	12 (4.2%)	17 (4.4%)	<0.05*
Normal (18.6–24.9kg/m^2^)	60 (58.3%)	122 (42.7%)	182 (46.8%)	
Overweight/obese (>25kg/m^2^)	38 (36.9%)	152 (53.2%)	190 (48.8%)	
**Ideal smoking habit** (Never smoked)				
Yes	85 (82.5%)	278 (97.2%)	363 (93.3%)	<0.05*
No	18 (17.5%)	8 (2.8%)	26 (6.7%)	
**Ideal physical activity** (≥150 min per week of moderate intensity activity and/or ≥75 min per week of vigorous intensity physical activity)				
**Yes**	76 (73.8%)	214 (74.8%)	290 (74.5%)	0.826
**No**	27 (26.2%)	72 (25.2%)	99 (25.5%)	
**Ideal diet** (≥20 total servings of fruits and/or vegetables weekly as the cutoff for ideal intake)				
**Yes**	9 (8.7%)	13 (4.6%)	22 (5.7%)	0.114*
**No**	94 (91.3%)	273 (95.4%)	367 (94.3%)	
**Alcohol consumption in the past 30 days**				
**Yes**	32 (31.1%)	65 (22.7%)	97 (24.9%)	0.093
**No**	71 (68.9%)	221 (77.3%)	292 (75.1%)	
**Clinical characteristics**				
**HIV viral load**				
≤ 50 copies	91 (88.3%)	250 (87.4%)	341 (87.7%)	0.804
≥ 50 copies	12 (11.7%)	36 (12.6%)	48 (12.3%)	
**Duration on ART**				
(Median and IQR) years	5 (3–10)	5 (3–10)	5 (3–10)	0.439

**Table 3:** Demographic, behavioral and HIV related factors associated with MetS

Characteristic	Univariate^a^		Multivariate	
	OR (95%CI)	P	Adjusted OR (95%CI)	P
Age (yrs)				
(Ref age=18–39)				
40–49	2.21 (1.12–4.37)	0.02	3.25 (1.80–5.84)	<0.001
50 ≥	3.92 (1.98–7.76)	<0.001		
Sex (Ref=Female)				
Male	0.89 (0.50–1.56)	0.68	0.88 (0.46–1.68)	0.70
Edccation Secondary and above (Ref=Primary level)	1.03 (0.59–1.78)	0.92		
Occupation, Employed (Ref=Unemployed)	0.67 (0.35–1.27)	0.22		
Ideal BMI^b^ (Ref=No)	0.18 (0.10–0.33)	<0.001	0.16 (0.09–0.30)	<0.001
Ideal Smoking habit^c^ (Ref=No)	0.87 (0.42–1.81)	0.71		
Ideal physical activity^d^ (Ref=No)	0.89 (0.51–1.55)	0.69		
Ideal diet^e^ (Ref=No)	0.36 (0.08–1.59)	0.18	0.48 (0.10–2.29)	0.36
Alcohol use (Ref=No)	1.7 (-/00–2.89)	0.05	1.55 (0.86–2.78)	0.14
Duration on ART (per 1 unit change)	1.01 (0.95–1.06)	0.78	1.01 (0.96–1.07)	0.56
Viral load >50 (ref≤50 copies/mL)	0.62 (0.26–1.43)	0.26		

a covariates significant at <0.2 were included in the multivariate analysis

b Ideal BMI: <25 kg/m^2^

c Ideal smoking: Have quit >12 months ago or never smoked

d Ideal physical activity: ≥150 min per week of moderate intensity activity and/or ≥75 min per week of vigorous intensity physical activity

e Ideal diet: ≥20 total servings of fruits and/or vegetables weekly as the cutoff for ideal intake

## Data Availability

The datasets used and/or analyzed during the current study are available from the corresponding author on request.

## Abbreviations

MetS: Metabolic Syndrome
ALHIV: Adult living with HIV
PLHIV: People living with HIV
CVD: Cardiovascular diseases
BMI: Body mass index
(NCEP ATP III): National Cholesterol Education Program Adult Treatment Panel III
ART: Antiretroviral regimen
CDs: Communicable diseases
NCDs: Non-communicable diseases
CTCs: Care and treatment clinic
INSTI: Integrase Strand transfer Inhibitors (INSTI)-
HDL: High density lipoprotein
LDL: Low density lipoprotein
SSA: Sub-Saharan Africa
HIC: High income countries
